# Exploring business models for managing uncertainty in healthcare, medical devices, and biotechnology industries

**DOI:** 10.1016/j.heliyon.2024.e25962

**Published:** 2024-02-07

**Authors:** Ehsan Javanmardi, Petra Maresova, Naiming Xie, Rafał Mierzwiak

**Affiliations:** a College of Economics & Management, Nanjing University of Aeronautics and Astronautics, Nanjing, China; b Faculty of Informatics and Management, University of Hradec Kralove, Hradec Kralove, Czech Republic; c Faculty of Engineering Management, Poznan University of Technology, Poznan, Poland

**Keywords:** Business models, Health-tech, Sustainability, Healthcare, Uncertainty, Innovation

## Abstract

The medical devices, biotechnology, and healthcare industries are closely tied to innovation, with companies relying on research and development and new product introduction. However, innovation activities like research and development can be costly, and commercializing new products faces complexities. Having effective business models (BMs) aligned with such innovation activities is crucial. This study delves into the intricacies of BMs in these innovator companies, emphasizing the pivotal role of effective BMs in navigating uncertainties and fostering innovation. This study conducted a systematic literature review to synthesize knowledge on BMs in innovator health-tech companies and compare models on dimensions like infrastructure, offering, customers, and finances. The review of 34 recent papers revealed 9 key BMs - open innovation, sustainable, dynamic, dual, spin-off, frugal, high-tech entrepreneurial content marketing, back-end, and product-service systems BMs. The analysis found open innovation, sustainability, and dynamicity as foundational models that can serve as a basis when combined with others. The paper unveils a tailored Dynamic Sustainable Business Model (DSBM) for Health-Tech, designed to integrate adaptability and sustainability, providing a framework for companies to leverage emerging technologies effectively. Additionally, a conceptual framework outlining 28 groups of uncertainty factors in BMs was developed to aid risk management in health-tech. The findings offer crucial insights for companies in health-tech industries, aiding them in managing innovation and value creation amidst a rapidly evolving landscape.

## Highlights

• Systematic review of 34 papers synthesized knowledge on business models in health-tech.
• Identified and categorized 9 major health-tech business models.
• Open innovation, sustainability, and dynamicity emerged as foundational business models.
• Developed framework outlining 28 uncertainty factors in health-tech business models.
• Proposed a dynamic sustainable business model for health-tech.

## 1 Introduction

Although the business environment is normally in constant flux, business-related transformations have recently accelerated; over the past decades, the business environment has undergone major changes in most industries. Technological advancements and the expansion of a large number of communication facilities among industries have increased competition on a global scale and have led to rapid changes in terms of methods of production and provision of services/products. The incessant competition in today's radically transformed world has urged companies to innovate novel ideas and pursue continuous development in all of their business-related activities, such as providing new services, proposing innovative and modern production methods, and managing their (sub)organizations and commercial transactions.

The emergence of new commercial activities helps to expand innovation, increase employment, and introduce positive aspects in the entire economic system while contributing to the system's efficiency [1]. Many researchers have acknowledged the impact of innovation on economic growth and development. The vital function of technology in economic development and social change has made the notion of “product development and innovation” the key variable in developing and emerging economies [2]. One of the significant fields in high-tech industries in the healthcare sector involves medical and biotechnological equipment. Nowadays, most industrialized countries actively support the progression of health-related innovations ranging from medical devices to biological and biotechnological equipment. Biotechnology and health-related devices strongly rely on science and innovation. Yet this creative field has faced a strong, yet high-risk, demand; the high risk associated with innovation and research and development (R&D) activities in this area has complicated the status of companies and firms engaged in these activities [3].

The healthcare industry, often referred to as the medical industry or health economy, is a comprehensive aggregation of sectors within the economic system that provides a wide range of services for patient care, including curative, preventive, rehabilitative, and palliative treatments [4]. This industry is crucial for the creation and commercialization of products and services aimed at the preservation and restoration of health and well-being. As one of the world's largest and fastest-growing industries, healthcare plays a significant role in the economies of developed nations, consuming over 10 percent of their gross domestic product (GDP). In 2021, U.S. healthcare spending alone grew by 2.7 percent, reaching $4.3 trillion, which accounted for 18.3 percent of the nation's GDP [5,6].

Within this vast industry, biotechnology stands out as a multidisciplinary field that combines natural and engineering sciences to apply organisms, cells, and molecular analogues for developing various products and services. This integration is pivotal in advancing healthcare through innovative treatments and technologies [7]. Similarly, the medical devices sector is an integral part of the healthcare industry. Medical devices, which range from simple tools to complex machinery, are essential for medical purposes. Due to their potential risks, these devices undergo rigorous testing to ensure safety and efficacy before they can be marketed [8]. The industry's importance is highlighted by its steady growth, with global sales expected to increase by over 5% annually and reach nearly US$800 billion by 2030. This growth underscores the medical device industry's critical role in advancing healthcare technology and improving patient outcomes [9].

According to the 2019 WHO report, there is still a major mismatch between demand and supply in the field of healthcare, while in many countries there is a deeply felt need for supply and the development of activities in this area [10]. An issue of concern is that although there is a high scientific potential in the field of medical/biotechnological equipment in many counties (e.g., especially those in the European Union), there is still no effective and practical system for transferring new solutions to markets to commercialize ideas and innovations [11]. According to research, it would be very unlikely for a company to pass through the implementation stage successfully; more specifically, the likelihood of success of an idea that goes all the way to the commercialization stage and is accepted in market is only 13% [12]. In the healthcare area, too, the investigations highlight that approximately 50% of the registered medical equipment inventions are never developed into medical products [13]. In most cases, a company may propose a practical innovation, although there are factors that negatively affect the company's performance at the implementation stage and sometimes the idea ends in failure [1].

Therefore, simply having a “good idea” for initiating a business cannot guarantee success but, in some cases, may even drag the company to bankruptcy. Plausibly, what is important is not just a good idea but the capacity to turn it into an innovative business model (BM) that converts technological opportunities into actual products/services, offer them in market, and finally generate value for the company and other beneficiaries. An effective BM can serve as a source of competitive advantage for companies in today's complicated and constantly changing environment; of course, a company can reach an appropriate competitive position only through a functional BM [14]. A BM, in its simplest configuration, is a method of doing business that helps a company to survive by earning income. Such a model clarifies how the company can generate value (a product/service) for which customers would be willing to pay [15]. A BM is an approach to doing business through which a company can sustain itself and gain profit [16]. A successful BM points to a better alternative beyond the existing choices while offering more value to customers and bringing back more profit to the company.

Of course, in cases where an inappropriate BM is used for the initiation of a business, the BM could keep the organization from accomplishing its goals [17]. As such, a BM can be regarded as a tool for consolidating technological development and generating economic value [18]. A well-structured BM should be able to receive some data about the target customers, the customer value, and the business procedure while explaining the underlying logic of the organization's economic activities and clarifying how the value sought by customers can be supplied at a reasonable cost [19]. Of course, constructing a BM encompassing all of the major channels of a business can serve as the factor of success, especially in the very first stage of establishing the company or developing a product [20]. The reason for that is that even the most original ideas will not be considerably valuable unless their originators mange to shape business models (BMs) capable of capturing the value of the idea or innovation [21]. The problem, however, is that there is little empirically documented evidence to help entrepreneurs realize which BMs they should adopt for their innovative commerce.

In addition, examining BMs in companies operating within the Healthcare, Medical Devices, and Biotechnology Industries is crucial for effectively navigating uncertainties. These industries operate in intricate ecosystems involving multiple stakeholders, including patients, healthcare providers, regulators, insurers, and investors [22]. Understanding these complexities is essential for adapting to uncertainties that can arise from changes in regulations, patient preferences, or technological advancements. Healthcare-related industries are highly regulated, and regulatory changes can significantly impact operations. Examining the business model helps companies prepare for potential regulatory shifts, ensuring compliance and minimizing disruptions caused by uncertainties in regulatory environments. Similarly, medical technology and biotechnology are rapidly evolving fields [23]. Analyzing business models allows companies to assess their adaptability to new technologies or changes in existing technologies, ensuring competitiveness and the ability to seize emerging opportunities. As patient needs and expectations evolve, business model analysis ensures that offerings remain patient-centric and aligned with emerging healthcare trends and patient demands. Uncertainties can disrupt resource availability and allocation. Business model examination aids in strategically allocating resources for research, development, innovation, and risk mitigation, ensuring the company's resilience. In conclusion, examining business models in the Healthcare, Medical Devices, and Biotechnology Industries is vital for effectively addressing uncertainties. It fosters proactive planning, innovation, risk mitigation, and strategic adaptation, crucial for navigating these industries' dynamic landscapes while delivering quality healthcare solutions and advancements [24].

In highlighting the complexities of business models within the Healthcare, Medical Devices, and Biotechnology Industries, the study uncovers a significant research gap. There is no comprehensive description of corporate behavior in these markets, especially in terms of how various dimensions of the business model (BM) interact. Obviously, there is a shortage of systematic and general studies dealing with how BMs help companies to administrate their products and what factors may affect the whole process. As a result, there is a need for describing and comparing companies’ BMs in this area, from the perspective of innovation process management and the introduction of new products. More importantly, businesses are complex, dynamic and uncertain systems due to multiple internal and external interactions. Medical devices, biotechnology and high-tech industries in the healthcare sector, as knowledge-based businesses, are involved in greater uncertainty. Empirical studies show that knowing the important causes of uncertainty increases the efficiency of companies' decision-making in dealing with uncertainty [25]. As a result, understanding the roots of uncertainty in this business will lead to a more realistic understanding and design of BMs. Since the aspect of risk and usual uncertainty of BMs in the companies in this field has not been considered enough; Therefore, at the end of the article, the authors will attempt to create a framework outlining the causes of uncertainty in business models and their impact on the business models of medical devices, biotechnology, and high-tech industries in the healthcare sector. The basic question in this section is what are the roots of uncertainty and risk in different parts of the BM of medical devices, biotechnology and high-tech industries?

Finally, this systematic review study aims to advance understanding of business models used in medical devices, biotechnology, and healthcare industries for new product innovation. The objectives are threefold: (1) synthesize current knowledge on BMs through a systematic literature review, identify and categorize major models used, and compare them based on key dimensions like infrastructure, offering, customers and finances; (2) contextualize and discuss BM specifications in medical/biotech markets and new product commercialization; and (3) develop conceptual frameworks - one on causes of BM uncertainty, and one proposing a tailored dynamic and sustainable BM for the health-tech industry. The goal is to fill gaps in empirical evidence on optimal BMs for companies commercializing new medical, biotech and healthcare products, providing them updated knowledge and practical frameworks to adopt BMs that align with innovation activities and address uncertainties. This will ultimately support successful innovation management and value creation from emerging technologies in these knowledge-based industries.

The research question, which probes into the roots of uncertainty and risk within the business models of medical devices, biotechnology, and high-tech industries, is pivotal to the study's aim of enhancing business model understanding in these sectors. These industries are characterized by rapid innovation and inherent risks, particularly in R&D activities, making it essential to explore how business models can effectively manage and mitigate these challenges. Addressing this question sheds light on strategic risk management and decision-making processes, crucial for navigating the dynamic environments of these sectors. Furthermore, the investigation enriches both practical and theoretical frameworks, advancing the comprehension of how business models should adapt and evolve in response to the unique uncertainties and risks present in these technology-driven fields.

In this study, the interconnected realms of healthcare, medical devices, and biotechnology are delved into, with their synergistic relationship being recognized. These sectors are intrinsically linked through a shared reliance on cutting-edge science and technology, driving innovation and addressing critical health challenges. Advancement in healthcare is often contingent upon the development of new medical devices and biotechnological breakthroughs, highlighting their interdependent nature. Furthermore, these industries are bound together by similar regulatory landscapes and market dynamics, impacting each other reciprocally. This interconnectedness is further emphasized by common stakeholders, ranging from healthcare providers to patients, who engage across these sectors, demonstrating their intertwined operations. The focus on these industries is chosen due to their position at the forefront of technological and innovative developments. Their high-risk, high-reward nature provides a distinctive view into how business models navigate uncertainties in an environment constantly evolving due to technological advances and regulatory changes. The exploration of these sectors aims to provide insights into how business models can adapt and succeed amidst the complexities and dynamism inherent in these crucial areas of societal and technological progress.

In the following, the study primarily describes the structured approach for the literature review, screening and analysis. The results section summarizes the findings, and categorizing nine major business models. The discussion section further compares the models across four dimensions of infrastructure, offering, customers and finances, noting open innovation, sustainability and dynamicity as foundational models. It explores business model specifications in the medical/biotech industry through sample case studies. It also proposes a framework outlining 28 groups of uncertainty factors in business models. Finally, it proposes a tailored Dynamic Sustainable Business Model (DSBM) for the health-tech industry that integrates sustainability, innovation and adaptability. Together, the results and discussion sections enable comprehensive analysis of business models for medical/biotech innovation and provide practical insights and frameworks for companies to successfully leverage emerging technologies. Finally, the conclusion summarizes the key goals, reiterates contributions, and discusses implications.

## 2 Methodology

This systematic review investigated various BMs and compared them in medical equipment, biotechnology, and high-tech industries in the healthcare sector. The selection of specific fields such as medical equipment, biotechnology, and high-tech industries for this study was guided by their critical importance within the broader healthcare sector. These fields are at the forefront of healthcare innovation, characterized by rapid technological advancements and significant impacts on healthcare outcomes. They present unique challenges such as navigating complex regulatory landscapes, managing high costs of research and development, and adapting to fast-paced technological changes. These aspects make them particularly relevant for a detailed exploration of business models, as they provide a rich context for understanding how organizations can effectively manage uncertainty, foster innovation, and adapt to evolving market dynamics. Furthermore, the focus on these areas addresses notable gaps in existing research, specifically concerning the application and effectiveness of business models in environments marked by high stakes and rapid innovation. By concentrating on these fields, the study aims to contribute valuable insights into the strategic management and operational dynamics of businesses operating at the cutting edge of healthcare technology.

The investigation and comparison of the BMs used in the innovative companies in this line of business followed four major dimensions and nine components (sub-sets): (a) Infrastructure: key activities, key resources, and partner network; (b) Offering: value propositions; (c) Customers: customer segments, channels, customer relationships; and (d) Finances: cost structure and revenue streams. The present systematic review sought to identify and compare the most frequently used business approaches in companies engaged in medical devices, biotechnology, and high-tech industries in the healthcare sector, and to find their methods of managing new products. To accomplish this, a four-phase verification process was conducted on the literature: (a) searching and selecting the relevant papers; (b) re-inspecting the papers selected; (c) certifying full-text papers to in terms of the inclusion criteria; and (d) scrutinizing and investigating the 34 papers that met the inclusion criteria.

The choice of a systematic literature review as the research methodology was deliberate and strategic, considering the complex and multifaceted nature of business models in the healthcare, medical devices, and biotechnology industries. This approach allowed us to comprehensively gather and synthesize existing literature, offering a holistic view of the current state of knowledge in these rapidly evolving fields. A systematic review is particularly well-suited for areas where interdisciplinary research converges, as it provides an unbiased, thorough, and reproducible method to collate and analyze diverse studies. This method ensures that the investigation covers a broad spectrum of perspectives, addressing the nuances and intricacies inherent in these industries. Additionally, given the dynamic and rapidly evolving nature of these sectors, a systematic literature review enables us to capture and integrate the latest research findings, ensuring this study remains current and relevant. This methodology aligns perfectly with the objective to provide a deep and comprehensive understanding of business models, effectively addressing the existing gaps and emerging trends within these critical fields.

During the initial half of 2023, a comprehensive search was carried out within the databases of Web of Science (WoS) and Scopus to explore the relevant literature pertaining to the research topic. The search process focused on papers published and indexed between 2014 and the first half of 2023 within these databases. A deliberate focus was placed on papers published from 2014 to the present, covering a comprehensive 10-year period. This duration was chosen to include a significant and recent era in the healthcare, medical devices, and biotechnology industries. The decade-long review facilitates a thorough examination of the evolution and trends in business models, achieving a balance between the latest advancements and adequate historical perspective. This timeframe is particularly pertinent as it encompasses a period marked by rapid technological progress and notable shifts in global healthcare dynamics. This period provides a robust foundation for the analysis of contemporary business models and their adaptation to emerging challenges. The selection of papers was facilitated using the keywords as detailed in Table 1.

**Table 1:** Paper distribution by property.

Keywords	Web of Science	Scopus
(Company OR Firm) AND (“Business model”) AND “new product"	242	268
(Company OR Firm) AND (“Business model”) AND “medical device"	22	24
(Company OR Firm) AND (“Business model”) AND “high tech"	118	107
(Company OR Firm) AND (“Business model”) AND “biotechnology"	63	73
(Company OR Firm) AND (“Business model”) AND Healthcare	105	154
**Total Publications (after removing duplicates)**	**524**	**598**
**Total Publications (Limit to English Articles or reviews)**	**324**	**336**
**Total Publications After Remove Duplicates in all data bases:** 434		

These keywords were selected because it was assumed that these key terms could effectively help to find the relevant papers and optimally direct the study toward its objectives. Keywords for the systematic literature review were selected through a combination of expert judgment and iterative refinement to ensure comprehensive coverage of relevant literature. The selection of specific terms such as “new product,” “business model,” “medical device,” “high tech,” and “biotechnology” was influenced by their frequent use in existing literature related to healthcare, medical devices, and biotechnology. Preliminary searches were performed to evaluate the prevalence and significance of these terms in scholarly articles. It was confirmed during this exploratory phase that these keywords effectively encompass a wide array of innovative practices and business models pertinent to the targeted sectors. The inclusion of the term “new product,” despite its broad nature, was deliberate to cover a range of innovations within the healthcare industry, thereby ensuring that the review did not miss relevant studies that might not specifically mention “medical devices” or “biotechnology.” The strategy behind this multifaceted keyword approach was to achieve a balance between specificity and breadth, capturing insights that were both directly targeted and peripherally relevant to the study's objectives. Because biotechnology was a sub-branch of high-tech industries in the healthcare sector, the term “high-tech” was also included in the search procedure. The keywords were searched exactly as they are mentioned above; the quotation marks were used to find the journals containing the exact key terms, because without the quotation marks a large volume of inconsistent results would be found, which could undermine the accuracy of the investigation. Based on the searches conducted, there were 1176 publications found on the databases, out of which 626 publications were indexed on Scopus based on keywords. On this database, too, the fields related to keywords”, “abstracts”, and “titles” were included in the search procedure. The publications found on WoS were 550 where the keywords focused on “TOPIC” and “all databases.” Table 1 shows the statistics of the publications based on the keywords searched on the databases.

**Fig. 1:**
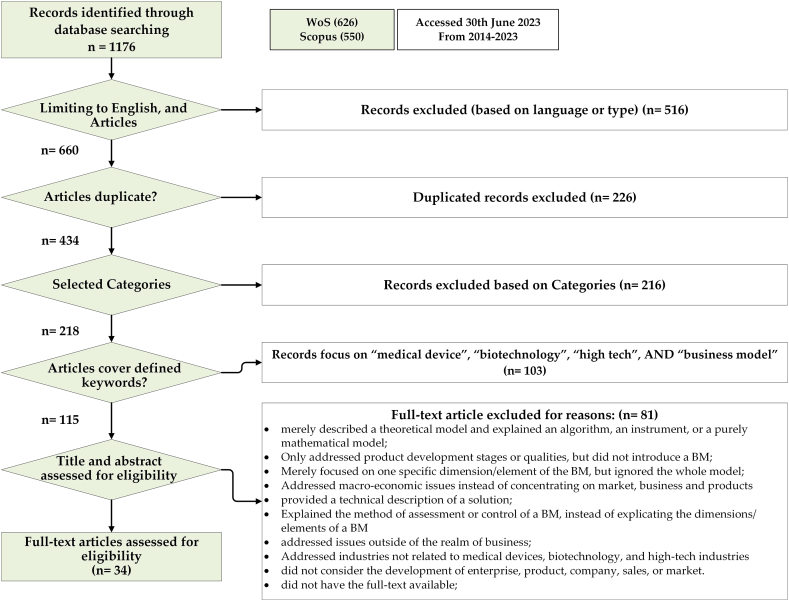
Publication search process according to PRISMA.

The study basically relied on keywords in the bibliographical options provided for searching. After the initial search, the repetitive records were omitted, as a result of which the number of the publications was cut down to 434 ones. Next, the titles, keywords, and abstracts of the remaining records were meticulously investigated to confirm their compatibility with the objectives of the present study. Some publications appeared to be relevant to the purposes of the study considering their keywords and titles, although upon further scrutiny it was found they were not in line with the scope of the study. Through the investigation, the publications irrelevant to the fields of medicine, biotechnology and high-tech industries in healthcare sector were omitted as well. After the irrelevant publications were removed in the initial stage, a total number of 218 works remained, although the number was reduced to 115 ones following a more meticulous investigation. The full texts of the remaining works were inspected (semi)manually based on the criteria mentioned in Fig. 1, and some papers which primarily seemed relevant were then screened. Following this final screening procedure, only 34 totally qualified papers were selected, which were thoroughly scrutinized and shaped the core of the analyses in this systematic review.

Fig. 1 illustrates the number of the papers and stages of the screening process. Primarily, the following data were collected from each of the papers: author(s), year, title, country/countries, the industry under investigation, objectives, methods, main finding, the type of BM used, and a brief description of the model. As a standard model confirmed by the scientific community, PRISMA (2009) used in this study for the search, selection and analysis of the publications; the reason PRISMA (2009) was used as the model and framework in this research was that it is regulated by an evidence-based minimum set of items in making reports of systematic reviews and meta-analyses. Fig. 1 schematically represents the process of searching, selecting and analyzing the publications found in this study (see RRISMA 2009) [26].

To ensure the rigor and reliability of the systematic literature review, a comprehensive quality assessment process was implemented, based on the criteria outlined by Kitchenham et al. (2009) [27]. This involved evaluating each selected study using four key quality assessment (QA) questions: (1) clarity and appropriateness of inclusion and exclusion criteria; (2) comprehensiveness of the literature search; (3) quality and validity assessment of the included studies; and (4) adequacy of the basic data and study descriptions. Each study was scored on these criteria using a standardized scale: Y (Yes) for explicit compliance, P (Partly) for partial compliance, and N (No) for non-compliance. This scoring procedure helped in objectively assessing the quality of the studies, ensuring that the review included only those papers that met high standards of research integrity and relevance. In cases of discrepancies or uncertainties in the quality scores, thorough discussions were engaged among the research team to reach a consensus. Additionally, outreach to the authors of the primary studies for clarification was undertaken whenever necessary. This meticulous quality assessment process underpinned the credibility and robustness of the systematic literature review, aligning with the standards set forth in the field of software engineering research.

## 3 Results

Following the detailed investigations, the filters employed, and the screening procedure conducted in this study, only 34 papers met the inclusion criteria. Table 2 lists the papers along with their specifications including the industry under investigation, the BMs used, and a brief account of the BMs proposed. The papers listed in Table 2 are sorted according to the year of publication. These studies addressed a wide variety of topics related to the BMs, especially in medical devices, biotechnology, and high-tech industries in the healthcare sector. Following the analysis of the selected papers, nine general business models were identified, along with papers exploring the various dimensions of these models. The open innovation BMs were the most popular ones in the studies selected. Following them, sustainable BMs were the second group of models addressed in the papers. This trend revealed that open innovation and sustainable BMs were more frequently utilized ones in the medical devices, biotechnology, and high-tech industries in the healthcare sector.

**Table 2:** Summary of studies.

Study	Country	Industry	Type of model (short description)
[13]	Canada	health technology industry	Spin-offs BMs: Examining three Canadian spin-offs
[18]	Finland	Healthcare technology	Open innovation BMs: Describing the main functions of the BM for the commercialization
[28]	UK, USA	Medical device	Single use BMs: The current BM in medical device manufacture was a hindrance
[11]	Argentina	Medical healthcare	High-tech-low-fee BMs: Presenting a new conceptualization on BM innovation that includes three dimensions: firm-centric, environment and customer-centric.
[20]	Poland	Biotechnology spin-offs	Spin-offs BMs: Identifying the BM components and related attributes of biotech spin-offs
[29]	Argentina	Biotechnology	Joint venture BMs: Presenting a business decision-making situation
[30]	Germany	SustainablySMAT	Sustainable BMs: Illustrating a new, environmentally sustainable value creation logic
[31]	UK, USA	Investment banks	Evolutionary and revolutionary BMs: examining how the level of dominance in firms
[32]	USA	“The Google of Healthcare”	Back-end BMs: 23andMe is back on the market as the first direct-to-consumer genetic testing company
[33]	UK, Portugal	High-tech entrepreneurs	The high-tech entrepreneurial content marketing BMs: Presenting the framework with five delineating elements
[34]	India, China	Medical and laboratory equipment	Frugal BMs: Investigating BMs for frugal innovation in the context of emerging markets. Investigating firms' ability for setting up value creation and value capturing mechanisms
[16]	Italy	EU Horizon 2020 “i3 project”)ICT(	Digital sustainable BMs: Investigating the role of digital platforms as facilitators for the techno-socio-economic impact assessment and the development of sustainable BMs.
[35]	Italy	Machinery, automation and transportation	Product-service systems BMs: Proposing a new integrated multi-step methodology for the selection and design of the most appropriate product-service systems Bms.
[36]	UK	Biotechnology	Open innovation BMs: Identifing two different BMs in Technology
[37]	Brazil	Second generation bioethanol	Open innovation BMs: In the comparative evaluation of the BMs that have achieved commercial scale production of second generation bioethanol
[38]	China	Production and Service	Open innovation BMs: Underscoring the importance of matching the BMs to open inovation
[39]	Finland	Digital hightech start-up	Agile BMs: Investigating alternative approaches under a time-constrained setting
[1]	Italy	Mobile application	Dynamic BMs: Starting from the lean BM canvas
[40]	Italy	ICT, pharma and biotech	Ambidextrous BMs: Examining impact of (a) the initial business model of a start-up, (b) the subsequent changes in the design themes and (c) the combinative effect of efficiency
[41]	Netherlands	General	Dynamic BMs: Focusing on dynamic approaches
[42]	Hong Kong	Software development	Dual BMs: High-technology small and medium-sized enterprises
[43]	Ireland	Medical devices	Collaborative and in-house NPD BMs
[44]	Korea	Medical device, healthcare	Sustainable BMs: Seeking to derive an activation strategy for medical device start-ups based on a priority analysis using the analytical hierarchy process (AHP)
[45]	Finland	Biomaterials, biotechnology	Circular bioeconomy BMs: How do small and medium-sized enterprises (SMEs) propose, create and deliver, and capture value
[46]	Brazil	Medical device	Circular BM: Identifying opportunities for circular business model (CBM) innovation
[47]	Brazil	pharmaceutical industry	Sustainable BM: Exploring eco-innovative business models using product life cycle
[48]	Germany	Medical device	Intelligence BM: Exploring how research prototypes can be turned into a product-ready state
[49]	Brazil	biotechnology	Lean BM: Investigating how biotechnology startups exploit opportunities
[50]	Poland	healthcare services	Presenting a new business model on the healthcare market
[51]	Spain	Private Healthcare	Examining the role of public resource desynchronization on business model sustainability
[52]	Russia	healthcare	Exploring the implications of digitalization and business model innovation
[53]	Netherlands	healthcare	Delineating the relations between business model efficiency and novelty
[54]	Mexico	Purified water	Combines the advantages of the Business Model Canvas to define the crucial functions of a business model with the service blueprinting capacity to represent service processes
[55]	Ireland	Biopharmaceutical	Biopharmaceutical business and inequalities in healthcare in low income countries

In the following sections, the findings are reported according to the BMs used. In doing so, the BMs and the findings of other researchers in the papers selected are broken into the four main areas: infrastructure, value proposition, customers, and finance.

### 3.1 Open innovation BMs

The concept of open innovation BMs, explored in 9 of the 34 papers selected for this study, plays a pivotal role in the healthcare sector, particularly in the development of new products within medical devices, biotechnology, and high-tech industries. Open innovation in healthcare is characterized by the collaborative amalgamation of internal and external knowledge streams to foster innovation [3]. This approach transcends the boundaries of individual organizations, advocating for a networked environment where ideas are freely exchanged and co-developed across different entities [56].

In healthcare, open innovation BMs are crucial for addressing complex medical challenges, as they facilitate the pooling of diverse expertise and resources. This model is particularly effective in tackling the escalating costs of R&D and the need for rapid product development cycles in healthcare. By enabling collaboration, even among competing entities, open innovation BMs contribute to the formation of standards and institutional collaborations that are essential in the healthcare industry [31]. In the healthcare sector, open innovation is a key driver for advancing medical technology and collaborative innovation, particularly in medical devices. This approach brings together various experts, including hospitals and biotech firms, to create more innovative solutions swiftly. Open innovation enhances healthcare R&D, integrating external research and technology for groundbreaking treatments [36].

A unique aspect of open innovation in healthcare is its dual approach of outbound and inbound innovation. Outbound open innovation involves disseminating internal knowledge outside the organization, leveraging external technological capabilities. In contrast, inbound open innovation focuses on absorbing external ideas and knowledge into the company, enhancing its internal knowledge base [43]. This bidirectional flow of information is critical in healthcare, where emerging technologies and patient care practices evolve rapidly.

The open innovation model in healthcare significantly benefits from collaboration with other entities in the product development process. Such collaborations provide access to essential skills and resources, allowing healthcare companies to commercialize innovative products more swiftly. In a rapidly changing healthcare landscape, this collaborative approach enables companies to adapt quickly to new technologies, reducing their reliance on fixed assets that may soon become outdated [18]. In healthcare, open innovation boosts productivity and resource management, streamlining medical solution development. Leveraging external expertise and cross-industry collaborations optimizes resources, cutting down R&D time and costs. Sharing facilities, technical knowledge, and funding through open innovation leads to more effective resource allocation and increased medical innovation productivity. Collaborations, especially in specialized fields like biotechnology, allow tackling ambitious projects unfeasible under closed innovation systems, speeding up breakthrough medical treatments and technology development [38].

Agility, a key component of open innovation, is increasingly being adopted in healthcare. This approach allows healthcare organizations to respond quickly to changes in a volatile environment, ensuring that their services and products align with current demands and technological advancements. This synergy enables healthcare organizations to quickly access and integrate external knowledge and emerging technologies. This dynamic response to healthcare needs and crises accelerates medical solution development and implementation. Agility in open innovation facilitates continuous and parallel commercial procedures, a crucial aspect for startups and healthcare companies navigating the unstable and rapidly evolving healthcare sector [39].

In summary, open innovation BMs in healthcare are not just about the fusion of internal and external ideas but also about implementing a flexible, collaborative, and agile approach to innovation. This model's significance lies in its ability to accelerate product development, reduce R&D costs, and adapt to the dynamic nature of the healthcare industry, ultimately leading to improved patient care and medical advancements.

### 3.2 Sustainable BMs

Out of the 34 papers selected, 9 ones specifically addressed BMs governed by sustainability. Recently businesses and entrepreneurs following social objectives have come to the fore. The notion of sustainable BMs today is normally seen as a competitive advantage. For this reason, sustainable BMs that underscore joint values and sustainable principles governing collaboration and innovation have constituted a significant line of research in the literature of BMs [55]. Such BMs incorporate social, environmental and commercial activities to create values for customers and society [57]. A sustainable BM must ideally emphasize sustainable values for customers and other beneficiaries, create and deliver such values, and capture economic value beyond its organizational borders, while preserving or re-generating natural, social and economic capital [45].

A business's value propositions can encompass social and environmental values, along with economic ones. Furthermore, during the value creation and delivery stages, sustainability can be explored in terms of renewable resources, technological development, sustainable innovations, interaction with accountable suppliers, and establishing sustainable consumption. Finally, capturing sustainable value involves a fair (re-)distribution of revenue between the beneficiaries [45,58]. The value created as a result of sustainable strategy is something other than the economic value added (issues such as environmental effects, saving raw materials through recycling, and wider sources of benefits for the society [30]. The important issue is that the fields of healthcare and medical equipment require a very long time for technology development, clinical tests, issuance of permits, insurance registration, and distribution to be finally commercialized, and because of this situation they need a sustainable strategy [44].

Most of the sustainable BMs investigated in this study addressed circular economy and issues such as recovery and recycling of the products. As the findings of D'Amato et al. (2018) showed, a significant proposition of sustainable BMs is concerned with circular economy, which underscores the improvement of productivity and the current production/consumption system's recycling capacity through reducing inputs, enhanced practices, re-use of waste, and recycling. In the areas of health and biotechnology, too, the principles of circular economy are incorporated into bioeconomy, as a result of which the notion of “circular bioeconomy” is proposed; this field addresses efficient, renewable biological resources [45]. Moultrie et al. (2015), too, dealt with “single use” BMs, trying to propose a solution for converting these models into sustainable BMs. Many medical devices are produced to be used only once, and they are disposed whether or not they are used for patients. This process generates a large volume of waste. Because on a daily basis a large proportion of medical waste (including numerous medical devices) are produced by healthcare and treatment centers worldwide, it would be necessary to change the BMs governing these fields in favor of sustainability [28].

Although in many cases it would be a better option to dispose or discard materials, there are circumstances in which it would be possible to re-use some devices safely. Recently organizations have emerged in the US that process single use devices; from this perspective, a value proposition must address how medical devices can be designed in a way they environment-friendly [28]. Innovation evaluation and BM development can prove to be difficult tasks, although information/digital technology can simplify this task as a tool in the infrastructures of a BM. Digital platforms in sustainable BMs can indirectly find the relevance of players that were previously irrelevant, while providing technical solutions that enhance mutual relationships. Digital platforms also seek to expand the network through technical solutions [3]. Generally speaking, it could be asserted that the framework governing suitable BMs is multifaceted and takes into account social and environmental dimensions, coupled with economic issues. In such BMs, contrary to other BMs, the goals are not purely economic. In terms of value propositions, suitable models try to consider three areas: economy, society and environment. They offer a value proposition that advocates both economic profit and social/environmental responsibility. Among customers, companies using such models are regarded as companies that do not merely prioritize profit and at the same time care about social/environmental concerns [55].

Offering sustainable value propositions, such BMs distribute products that are less harmful to the environment, can be recycled, and even reduce demand for consuming hazardous goods on a social and environmental scale. Evidently customers of such companies, besides the general public, are citizens who are concerned with the environment and social values. As an instance, nature-friendly biotechnological products with a faster recycling process may be more expensive than ordinary products, but many environmentally concerned customers tend to buy such products. Moreover, in terms of infrastructure and value creation, a sustainable BM is characterized by a consideration of safety on corporate, social and environmental levels, the use of recyclable materials in the production process, the use of bio-base materials the production process, and the implementation of processes in the production supply chain that enhance sustainable advantages. In the process of capturing value, too, reducing environmental effects and considering social responsibilities, beyond increasing revenue and profit, are among the objectives and indicators of a sustainable business [28,45].

### 3.3 Dynamic BMs

BMs belong to the class of dynamically complex systems, which is a quality that makes it difficult to analyze and predict them. The large number of factors effecting various aspects of BMs, the complicated relationships between the factors of such systems, and the role of humans as the main players in these systems contribute to the high complexity and uncertainty of them. Given these characteristics, today researchers have shaped a specific line of research that deals with the dynamic aspects of business, particularly with explicating dynamic BMs [3].

Among the papers selected in this study, 3 ones specifically addressed dynamic BMs in the industries under investigation. The transformational or dynamic approach views a BM as a conception or tool used to overcome changes and concentrate on innovation in the organization or even in the BM itself. Today the dynamicity of a BM is an overriding notion because commercial technologies and the market in which high-tech companies work undergo changes over time. The framework of the dynamic BM captures the internal and external changes of the company by inspecting trends within a timeline, highlighting the relationships between the various components of the model. Adopting a systemic view, such BMs investigate different elements in a BM and try to increase a business's coordination with various sources of change in different business-related areas. Such changes may include environmental changes, customers' taste, competitors, technology and the like [41].

Dynamic BMs are called *dynamic* because they incorporate the element of time and consider temporal changes, as well as the mutual relationships between the elements of the model. One could argue that a dynamic BM involves the capacity to *sense* and shape “opportunities and threats” and to accomplish opportunities; to maintain competition, it reinforces, combines, protects, and if necessary, re-configure (in)tangible assets of the company. A company with a high rate of dynamic capabilities can quickly implement, examine, and refine newly revisited BMs [41]. A dynamic BM must formulate a customer relationship strategy and assessment measures.

Clearly, every customer segment exhibits its specific customer needs. Detecting customers and their needs is the cornerstone of a BM because changes in needs will lead to changes in value propositions, which in turn transform the infrastructure of the BM and the ways value is offered to customers. To explain types of customers, five categories have been suggested in the light of a product's lifespan. First, there are innovators who are the customers of a company's product development stage and are usually young people who have high education levels, tend to take risk, have access to financial resources, and have technical skills and a high rate of information. Second, there are early adopters who participate in the customer introduction stage; early adopters are skillful in motivating themselves and the early majority to accept a new product/service, making it possible for the company to increase its market share [3].

The third group are early majorities who are customers of the product growth stage. They hold a high degree of relationships and have a situation favorable for leadership, and resort to consultation to reach a new idea. The fourth category is called the late majority in which customers are usually pessimistic and outdated and have limited access to financial resources. At the maturity stage of the product, they become customers of the company. The fifth category includes laggards who are secluded/suspicious, have poor social relations, and are slow decision-makers. Identifying customers at every stage of the product's lifespan helps the company to modify its strategies according to each category of customers; in fact, this is a factor that lies at the core of a dynamic BM. Therefore, the factor of time should be taken into account in the organization from the initial stage. Adapting to market needs enables the company to realistically identify and follow the changes in customers' needs; this strategy can guarantee the source of revenue in time [1]. Furthermore, a dynamic BM must include important environmental variables that affect the BM variables, while considering the effect of the company on the business environment. A dynamic business framework must encompass the company's internal variables, the external environment's variables, and the variables that may change as a part of strategies or the BM as well as their interrelationships. The ability to recognize and assess mutual relationships and possibly existing causal relationships among the variables represents one of the foundational aspects of dynamicity. Working in a network, companies develop, produce, supply and hold products/services that are related to each other.

As time passes by, different players and network links contribute to the growth or degradation of advanced companies. Having knowledge about these mutual relationships enables managers to identify changes associated with the environment more quickly and easily. The other characteristics of dynamicity are adaptability, the correction of the mutual relationships, and the BM's specific function over time [41].

### 3.4 Dual BMs

Due to the complexities of commerce, today many companies simultaneously manage different business areas with different management goals. Dual BMs refer to a condition in which a company simultaneously pursues two strategies or two conflicting/incompatible organizational objectives, both of which are equally significant and excellent [34]. This condition is also called “organizational ambidexterity.” Metaphorically speaking, it could look like flying a plane while repairing its wires. This situation shows the company's capability of simultaneously making explorations and exploitations for the purpose of competing in mature technologies and markets where productivity, control and gradual improvement are valuable.

This condition also makes it possible to compete in new technologies and markets in which flexibility, independence, and experience are necessary. In today's business world, ambidexterity is an essential for success. This is especially true of technologically advanced enterprises that have no alternative but to use their merits to gain short-term commercial advantages, while they seek to achieve new merits for long-term success [40]. More specifically, small- and medium-sized companies that rely on advanced technologies will have to make distinct innovations to be ahead of their competitors and build advantage for themselves. At the same time, they must be efficient as well, because their economic foundations are not as robust as those of large organizations [42].

Findings suggest that ambidexterity in a BM can have a negative significant impact on the growth of startups, and that higher rates of ambidexterity could even damage the growth of startups. In the initial stages, ambidexterity tends to leave a negative impact. Afterwards, however, when the startup reaches maturity, ambidexterity can have a positive economic impact. In fact, pursuing conflicting objectives right after a startup has been established can impede its growth and hold its development. In contrast, in the subsequent stages when the startup has established its organizationally required procedures, has clarified its market position, and has examined its revenue models and cost structure, ambidexterity may release all of the potential of the startup and accelerate economic growth [40]. Deeper cooperation with partners possessing technology enables a startup to develop its internal capabilities, such as absorptive capacity, knowledge production, and creativity in its processes. A higher degree of cooperation, along with increased capacities, will ultimately accelerate product innovation and new approaches in the field of marketing. Findings suggest that productivity and novelty can co-exist in a BM. Specifically, a BM can follow efficiency but at the same time advocate novelty [42]. Dual BMs rest on two central facets: concentrating on efficiency and offering innovation. In terms of efficiency, the main objective is to develop technology to strengthen distribution channels and to accelerate the product innovation process. Innovation practices, from the perspective of efficiency, bring about innovation in processes for the purpose of creating and capturing value. Similarly, in terms of innovation, collaboration with foreign parties in product innovation, using third parties’ standard technologies in industries, can prove to be a significant goal. Incremental innovation reflected in the value proposition (e.g. product/services innovation) could also lead to development of the innovative approach [40,42].

### 3.5 Spin-off BMs

A spin-off is an operational strategy used by the company to establish a division of the parent company. A spin-off is part of a company/organization that has become an independent unit, using the assets, employees, intellectual property, or existing products of the parent company. To compensate for the loss of equity, shareholders of the parent company receive equal shares in the new division. Meanwhile, shareholders can buy or sell their shares independently from either of the companies, which is an option making investment in such companies more interesting. The reason for this is that potential share buyers can invest parts of the business that appear to be growing more than the other parts. A spin-off typically refers to a new, independent entity formed from a part of an existing organization, inheriting assets, intellectual property, or products from the parent company. However, the spin-off business model extends beyond this structural concept, encapsulating the strategic, operational, and innovative frameworks that these entities adopt to establish and grow their presence in the market. Unlike the mere structural separation implied by a spin-off, the spin-off business model is characterized by its approach to leveraging the unique assets and capabilities inherited from the parent organization for commercial success, particularly in the fields of healthcare and biotechnology [59].

Spin-off business model embody a blend of innovation-driven strategies and operational practices that are essential for the success and sustainability of spin-offs in these dynamic sectors. Today academic spin-offs have a considerable function in the commercialization of ideas. Most universities mainly focus on basic research and usually lack the effective commercial apparatus for their R&D results. This gap in the transfer of technology from basic research to commercialization can be filled by academic spin-offs that can transfer the technology of universities and research institutions to industrial companies [20]. University spin-offs normally lack the specialized commercialization skills in terms of innovation, while their markets face serious ambiguities as they have no image of how to create value through their products. This issue further underscores the significance of proposing a BM governing university spin-off. More specifically, spin-off technologies in the field of healthcare require well-designed BMs. The importance of such BMs inspired 2 papers to particularly explore them. Spin-offs in healthcare and biotechnology often originate from academic or research settings, bringing with them substantial intellectual property. Their business models are heavily focused on leveraging this intellectual capital, developing proprietary technologies, and navigating the path from research to commercialization. Given their origins and innovative focus, spin-off business models typically involve unique funding strategies. This includes securing venture capital, government grants, and partnerships with industry players to support their R&D efforts and market entry. These business models are designed to effectively enter and grow in highly specialized and competitive markets. Spin-offs often target niche areas within healthcare and biotechnology, exploiting their innovative edge and specialized knowledge. Also, integral to spin-off business models is the establishment of strategic partnerships. These can include collaborations with other research institutions, industry players for product development and scaling, and academic institutions for ongoing research support. In addition, a critical component of spin-off business models in these sectors involves navigating complex regulatory landscapes, ensuring compliance, and adapting to regulatory changes, which is vital for market success and patient safety. Meanwhile, Spin-offs are often more agile than their parent organizations, allowing them to quickly respond to new research findings, technological advancements, or changes in market demand. This agility is embedded in their operational model, ensuring that they can capitalize on new opportunities or pivot in response to challenges more swiftly than larger, more established companies [59]. By examining these distinct features, it becomes clear that the spin-off business model in healthcare and biotechnology is more than just a structural change from a parent company. It represents a comprehensive approach to innovation, funding, market entry, collaboration, and regulation, all of which are crucial for navigating the unique challenges of these industries.

Key transition periods for spin-offs include initial idea development, technology application, and eventual commercialization. Strategic partnerships, including collaborations with well-established companies and research institutions, play a crucial role in reducing business risks and facilitating market entry [13]. International collaborations and experiences on boards of directors also significantly contribute to successful commercialization in global markets. Financial stability, often bolstered by grants and investments, is crucial, particularly in high-risk R&D environments [20]. In summary, spin-off business models represent a comprehensive approach, integrating innovation, funding, strategic collaborations, and agile operations to navigate the unique challenges of the healthcare and biotechnology industries [13].

### 3.6 High-tech-low-fee BMs

One of the new trends in innovative BMs tries to provide medical and health-related services to the bottom-of-the-pyramid consumers. The framework of these BMs rests on the traditional idea of innovation, social profit equation, the general environment, labor, and end users, as well as the dynamics among these factors. Implementing a BM for the classes in the base of the wealth pyramid demands a high degree of cooperation with customers, suppliers, distributors, and commercial partners in the different “structural blocks” of the BM. The main approach in such BMs emphasizes a customer-oriented view, instead of a product-oriented or company-oriented one. In doing so, companies must shape a horizon of understanding that joins both non-profit organizations and social leaders (non-market players). In other words, this process involves collaboration and partnership with small-scale local jobs or civil organizations that are trusted or supported by local consumers; through this process, they can connect large companies to previously ignored markets or small companies to larger markets. Enterprises, too, must create value propositions that take into account consumers’ cultural and psychological tendencies, their general behaviors, and life conditions.

To accomplish these purposes, especially in the field of health, removing intermediaries (medical treatment insurance companies), inexpensive practices, outsourcing services, or a strategy for low-income or low profit-margin conditions but in large quantities (annual membership card costs) can serve as factors that offer value to customers and gain profit [11]. Another sub-set of such models is called frugal innovation BMs, which can help to implement low-cost high-tech BMs. Today, frugal innovations have activated new and unprecedented applications, generating new markets. There is a threefold value proposition in frugal innovations in the area of healthcare industries, and that companies would have to constitute compatible mechanisms for creating/capturing value for frugal innovations [34]. More specifically, to successfully compete in the segments of emerging markets, companies must come up with resource-constrained innovations and BMs that can create a high value against low costs. To succeed in new low- or medium-level markets, companies (particularly western ones) need to develop capacities for resource-constrained innovations and BMs. Frugal BMs also pursue a valuable proposition by not only reducing costs and lowering prices for customers, but also by proposing solutions that encourage customers to pay the price. The value proposition in these BMs must work according to financial cost-effectiveness, along with innovation, for customers. In these models, the general purpose is to create innovations that deliver the value to customers/beneficiaries at lower costs.

Target customer segments in these BMs in the area of health can increase productivity of the healthcare system, address operational requirements, increase the accessibility of clinics and doctors, and suggest solutions for patients' emergency needs, accessibility, and reasonable prices. To achieve frugal value propositions, companies optimize costs in every value creation to respond to the demanding cost/operation requirements of their resource-constrained customers. Meanwhile, companies’ product development activities focus on innovative and creative applications which are regulated by established technologies compatible with customers specific needs [11].

### 3.7 High-tech entrepreneurial content marketing (HIT-ECM) BMs

HIT-ECM BMs especially underscore marketing as a tool for value creation in high-tech companies. Of course, in small-sized technology-governed enterprises, marketing is differently investigated because entrepreneurs in the area of technology must adapt themselves to the short lifespan of technologies while commercializing their solutions. On this account, BMs that specifically focus on marketing issues are very important in this area. Marketing transformations have brought about new practices for doing business, a new logic for enterprises, and new ways of creating value. Content marketing can complement the value-creating performance of a company's BM, which is the same as the value created for technology users. Mansour and Barandas (2017) contend that HIT-ECM BMs are composed of five dimensions [33].

HIT-ECM is a mode of content marketing in unpredictable environments in which high-tech entrepreneurs rely on their capabilities and resources to innovate their marketing strategies and BMs. This process is accomplished by getting engaged in a dialogue with customers and by creating measurable value and content. Today, companies must regard mass media as unifying strategies that direct consumers' experiences. Content marketing is considered to be a comprehensive, systematic design of a marketing program. This type of marketing underlines the entrepreneurship value of the company's BM and links it directly to the creation of “suitable content” for all beneficiaries throughout business operations. Overall, it could be observed that such BMs try to identify customers, find them in their social networks, and publish optimized contents to attract customers through content marketing and to be in contact with them. Such BMs essentially emphasize marketing as a core for developing customer segments, revenues, products and even relationships with key partners [33].

### 3.8 Back-end BMs

*Back-end* refers to a proportion of activities conducted in a business that are rarely visible to people or customers. A company using a back-end BM has two value propositions: a value proposition offered to general customers, which shapes the *front* side of the business; for instance, online genetic test devices represent an innovation that enables customers to simply conduct some elementary medical examinations at home. Such an option encourages ordinary customers to buy the product. In such models, sometimes the company does not gain any profit from the value proposition because it tries to disseminate the product and gain profit through the back-end of its business. The back-end may include the main partners, major activities, or main resources. Put simply, the back-end side in a business encompasses any aspect not visible to customers. A good example of companies using this model in the area of health is “23andMe”, as the first company to directly sell genetic test equipment to consumers; the company's model involves two general components in its BM: first, the front-end business, which sells personal genetic tests online; and second, the back-end BM, which is one of the most comprehensive private genetic databanks in the world. Since 2007, 23andMe has offered inexpensive products to customers with requests for genetic analysis. This product uses a large amount of data of the consumers and sells them to gain profit, and for this reason it can be called the “Google” of personal healthcare [32].

### 3.9 Product-service systems (PSS) BM

Today's transition from traditional product sales BMs to new PSS can prove to be an opportunity for industrial companies to generate revenue and make competitive advantage. Faced with commoditization, reduced profitability, and customers with complicated needs, an increasing number of production companies transform their value propositions (from selling goods to suggesting solutions) to offer a competitive advantage, increase incomes and margins, meet customer satisfaction, and attract more customers. In fact, this transition demands a foundational change in the way value is created and delivered and the way customers and beneficiaries are treated [54].

PSSs are BMs that make it possible to consistently deliver products and services. In simple words, PSSs, contrary to the traditional emphasis on products, offer a combination of products and services. To create value for customers, such BMs move beyond mere product sales as the only operation of companies [35]. The motivation behind PSS BMs, which are regulated by product-services systems, arises from the fact that producers can no longer compete by merely producing and selling their quality goods [60]. Investigations into the impact of PSS BMs on the innovative behaviors of companies have clarified three determinants: (a) product ownership will not be transferred to customers, and the company remains the main agent offering products/services; (b) when a product/service is offered, products are used as tools to offer services, and not just as useable goods; and (c) the trading profit of product-service companies is remarkably different from that of companies that merely develop, produce and sell their goods [49]. The value proposition of such BMs mostly rests on the service offered by the product, not on a mere product in itself. Changing mental models must be excised in companies following this BM so that they can help consumers to see services as job opportunities and as potential resources for creating value [54].

Furthermore, to enhance customers' awareness of a new value proposition, a sales channel must be constituted to directly offer services. A company utilizing PSS BMs may earn revenue from units-of-service (or product performance) offered to customers. For instance, a possibility is to consider the frequency of a customer's use of the product. In the field of medical devices, a product may be used to conduct a medical test and the customer has to pay for each time the test is administered. Another mode of service provision may promote a strategy through which people who cannot afford to buy a product can have access to services instead. In such a situation, offering joint services of a product can generate revenue for the company [35]. Generally speaking, there are three types of PSSs. In product-oriented models, the provider sells the products while offering some extra services, such as repair, maintenance, consultation, and education. There are also models that work according to the time during which the provider preserves ownership but sells the tools for performance or operation, such as leasing, renting, subscription, and swimming pools. The third type includes result-based models in which the provider sells the results gained out of a product [54].

The potential advantages of this BM are clear; continuous revenue and long-term relationships with customers can be major motivations for using this BM. Moreover, PSS BMs have the potential to improve the environmental performance of the company because it is argued that service companies are highly motivated to internalize the external issues in the lifespan of their products, because their revenue and environmental advantages are positively correlated. The economic advantages of PSS BMs are better ways of meeting customers' needs, having stronger relationships with customers, creating distinction, increasing revenues, discovering new markets, making faster responses, having access to services data, reducing responsibilities of ownership for customers, improving technologies, reducing risks, and reducing costs of the product's lifespan. Such models can also engender specifically social advantages mainly through increasing the number of jobs [60].

## 4 Discussions

This section addresses four distinct topics. Primarily the BMs extracted from the papers will be compared and elaborated on from different perspectives, and their advantages, disadvantages, and business foci are explored in the field of medical devices, biotechnology, and high-tech industries in the healthcare sector. This process of comparison and investigation of the BMs relies on the components of a BM. Of course, each company may utilize several BMs in its different sections, and there might be a BM composed of several other BMs. For instance, a BM may work according to open innovation or sustainability, while another BM may be regulated by sustainability and dynamicity at the same time. The BMs are separately explored in this section to enhance the understanding of their dimensions/specifications, and not because of any inherent conflict between them. It should also be noted that in many studies spin-offs fall under open innovation models [48]; yet in the field of medical devices, healthcare, and biotechnology, as well as in innovative companies and start-ups, such BMs are particularly important and that is why there are considered to be a separate category of BMs.

In the second section, the BMs are investigated in the market of medical devices (biotechnology). In doing so, case studies dealing with medical devices or biotechnology in the papers selected are separately and specifically explored. A brief account of legal conditions specified in each case study, the type of financial supplies, target groups, customers, and type of products proposed in such models are elaborated on and compared. Table 3 provides a summary of the comparison of the papers under investigation, along with the most outstanding and significant specifications of each BM. In the third part, the dimensions of uncertainty in business models are addressed. This analysis is crucial for understanding how uncertainties influence BM dynamics, particularly in industries characterized by rapid innovation and change. Lastly, the fourth section introduces a DSBM for the Health-Tech industry. This model is a novel proposition that encapsulates the findings and analyses from earlier sections, providing a strategic approach that integrates sustainability, innovation, and adaptability. The DSBM is designed to manage the unique challenges and uncertainties prevalent in healthcare, medical devices, and biotechnology sectors effectively.

**Table 3:** A summary of the comparison of the BMs.

Type of model	Infrastructure	Value proposition	Customers	Finances
Open innovation BMs	- Cooperation and partnership with companies, research institutes and universities to outsource various parts of a project - Create alliances, buy scientific services, invest in other companies, and use external knowledge networks - Buying or selling the intellectual property	- Quality, “timely” delivery, differentiation, speed, accuracy, within the budget set and lean innovation	- Companies that want to outsource their R&D and seeks innovation and efficiency in a product. Including global pharmaceutical companies, regional distributors, patients, professional hospitals, doctors and governments	- Usually because of the project nature of the work, revenue will not be repeatable. - If innovation is successful, revenue can sustain a sales value stream for a long period
Sustainable BMs	- Using recycled materials - Developing hybrid business - Adopting innovative production process based bio-based material	- shifting from a consumer to a user logic - products using bio-based renewable material	- Market segments that are more concerned about the environment and society	- reducing costs, waste and virgin material use and minimizing enviromental impact
Dynamic BMs	- Designing feedback loops from the environment, competitors, and customers to get information - Designing agile processes	- The value proposition is not static, but it has a life cycle and changes in nature and value	- Customer segments can change over time	- revenue and cost sources are dynamic
Dual BMs	- Innovation in Processes and methods - Collaborating with other institutions and companies - Trying to increase efficiency and innovation at the same time	- Multiple value propositions based on innovation and efficiency	- Customers looking for incremental innovations along with efficiency and cost savings.	- revenue sources from innovations have higher risk but instead efficiency sector, support innovative financial needs
Spin-offs BMs	- Experienced managers Research teams - “star-scientist” Experienced manager	Entails both clinical and economic value for physicians	- Part of society seeks innovative value propositions	- Research partnering - Cost advantage due to research cooperation with university
High-tech-low-fee BMs	- Low-cost raw materials - Global sourcing for critical parts - Low-cost production - Decomposition of multipurpose machines into a focuses single purpose device	- Affordability and access to healthcare at low costs - Easy to use for rural general practitioner - Preventive screening	- Access to customers through new additional sales units for rural areas in Emerging markets, channels via distributors and direct selling	Cost minimization in each step of value chain and income is obtained by Product sales, Pay-per-use, Leasing, Software as a service.
The high-tech entrepreneurial content marketing BMs	- Engaging in discourses with customers through value-creating and measurable content through content marketing	- The approach is based on presenting a unique image that is independent of the value of the content	- Customers who are somehow active in social networks and social activities	- The cost of content marketing, especially on social networks, is the main difference between these models and others
Back-end BMs	- Create infrastructures to create value from the back end activities include big databases of front end customer's information	- Providing a value proposition based on company support activities to back-end customers	- Clients include patients, healthcare applicants for medical tests applicants, research organizations, governments, ministries and universities	- Back-end is generally the main revenue and profit making process - Main revenue is generally derived from the support activities
Product-service systems BMs	- Products with delivering part of their performance in the long term - Capture and use data generated by customers	- The goal is to deliver the performance of a product, not the product itself	- Customers include people who prefer to buy a product based on their usage	- Revenue is generally derived from the service units or performance of the product

### 4.1 Comparing and investigating the BMs

#### 4.1.1 Infrastructure: key partners, key resources, key activities

Because in medical devices, biotechnology, healthcare, and biotechnology, as sub-fields of a high-tech industry, competition is increasingly regulated by knowledge, the infrastructures of the BMs dealing with this industry remarkably rely on knowledge-based production practices and the commercialization of the knowledge produced [46]. Open innovation BMs, which were most frequently found in the papers selected, base their value creation process on collaboration and partnership. Collaboration is viewed as a source of value creation and as the main trajectory of product development and commercialization. In such BMs, companies active in one industry, those active in several completely different industries, and even competitors may have cooperation with each other. Collaborating companies in such BMs contribute to the development of research and innovation projects, by collectively providing the resources [29]. The role of the company, depending on the type of activities, may involve fund raising, contributing to study designs, obtaining permits and intellectual property rights, developing the network through attracting new partners, and managing the network [36]. Infrastructural activities conducted to create value in inbound open innovation BMs, especially in the area of new products in the healthcare and biotechnology industry, may include alliances, buying scientific services from companies engaged in various fields, payment for product use rights or buying intellectual property rights of other partners’ products, investment in other companies and institutions, and using external knowledge networks.

One of the common collaboration models in open innovation BMs involves selling/buying the use rights or intellectual property rights of innovative products. In the healthcare field, a company most often conducts innovations or experiments in terms of a medical device or a pharmaceutical, but the company is not capable of supplying the innovative product for an international market. In such a situation, the company may delegate the product use rights or product sales rights to another company that is capable of undertaking mass supply. In such a case the innovative company that sells the ownership of the product or its use rights is regarded as an outbound open innovation company, and the company buying it is seen as an inbound open innovation one [47]. In BMs governed by sustainability, what is important in the infrastructural section is the *result* of the activities, not the trajectories leading to the result [47].

What basically distinguishes dynamic BMs from other BMs lies in the foundational belief that the environment, costumers, competitors, and any other factor affecting a BM undergo change over time, while everything is regulated by dynamicity [41]. From the dynamic perspective, the infrastructures of a business are designed and structured in a way that they can rapidly change and update themselves by receiving feedbacks from environmental parameters [1]. Dual BMs, in terms of their infrastructures and value creation, equip corporate resources to achieve two goals: efficiency and innovation. Efficiency normally tries to develop technology to reinforce distribution channels and increase the pace of the product innovation process [40,42].

Contrary to the other BMs, in spin-off BMs the key resources are neither materials nor tools, but renowned scientists, faculty members, inventors, and experienced system managers. More specifically, in the field of medical devices and biotechnology, knowledge serves as the basic key resource, as a firm is initially established based upon a new mode of knowledge. In such business systems, activities that prolong the survival of the company include the attraction of financial resources from governments, investment institutions, and knowledge commercialization grants from institutions. Moreover, one of the basic strategic elements of firms’ growth in spin-off models is the reinforcement of their intellectual capital value [13,20].

In frugal BMs, or *high-tech-low-fee BMs*, the main objective of the value creation section is to reduce costs while maintaining a high level of innovation and technology. Some of the infrastructural measures taken in frugal BMs to reduce cost and provide a high-tech level of functioning are selecting partners and transferring the production process to countries that offer lower production costs, using inexpensive raw materials, using global sources for critical parts, utilizing digital and software technologies to reduce errors and loss, decomposing multipurpose machines into a focused single purpose device, and establishing collaboration and participation with local small-sized jobs or civil organizations supported and trusted by consumers [11]. Meanwhile HIT-ECM BMs specifically focus on marketing as a basic infrastructure for value creation in high-tech firms. The most distinctive difference of infrastructural activities in such BMs is the generation of processes that help the company to purposefully introduce innovative products/services to customers [33]. Contrary to most BMs, back-end BMs rely on their infrastructural processes as their main source of value creation. These BMs make profit not because of the products/services they offer to customers, but because of their *support processes* managing delivery to customers. Thus, in such models, infrastructures are designed in such a way that companies can make value/profit by creating value for a second category of customers who are mainly third parties or even governments [32].

Finally in new PSS BMs, infrastructural processes focus on both services and production because value is created through a combination of products and services. In such a case, infrastructures are not merely models of production or services, but a combination of both. Designing a product by considering the prospect of a service represents an important activity in successfully implementing such BMs [35].

#### 4.1.2 Value proposition

The second element addressed in a BM is the value proposition, which is the key link between the infrastructure and customers and one of the basic motivations for customers to pay for a given product. In open innovation BMs, the value proposition functions based on quality, timely delivery, distinction, speed, accuracy, affordability, and uniqueness of the innovation and services offered. The service includes proof of concept, final design, testing and verification [43]. On this account, the value proposition may be offered as an option that increases efficiency and reduces transactions/coordination costs, or as a strategy emphasizing the novelty of the service/product [38]. Yet, in sustainable BMs, the business value proposition, besides economic value, encompasses social/environmental value. For this reason, part of the value proposition in sustainable BMs may advocate a reduced rate of resources, less production of waste and pollutants, recycling waste to produce more valuable products, the production of bio-based products with recyclable materials, an attempt to reduce consumption by providing re-useable products, and reducing energy consumption [45].

Dual BMs simultaneously emphasize several value propositions regulated by innovation and efficiency. Spin-off BMs, by identifying “covert” value propositions in new technologies, seek to find forward-looking customers who appreciate technology-based value propositions. The value proposition in such models specifically works according to innovation, technology and uniqueness. In frugal BMs, the value proposition involves several dimensions; one of them concentrates on cost reduction and consequently lower prices for each unit for customers. The other dimension seeks to find solutions that increase customers’ tendency to pay for costs.

In frugal BMs, the value proposition for the customer represents a cost-effective solution that brings about a high degree of value. Moreover, value propositions in high-tech-low-fee BMs delivers a high value for end customers (e.g., patients) and B2B customers (e.g., doctors and small hospitals), while reducing the general cost of the entire health and treatment system. HIT-ECM BMs tend to propose value propositions through content marketing. Products are independently offered and the uniqueness of the content value is the basic component of such models [33]. Value propositions in back-end BMs are composed of several aspects. First there is the value proposition offered to customers and encourages them to use services/products. There is also another value proposition on which the main revenue of the company rests and is usually offered to special customers who perform covert or support activities [32]. Finally in PSS BMs, value propositions are shared based on the services offered or a solution provided. PSS BMs, compared to traditional product-oriented models, offer a combination of value propositions regulated by the products/services specifications, and they usually seek to provide the *function* of a product, not itself [35].

#### 4.1.3 Customers: customer segments, channels and communications

In the BMs addressed in the publications, customers included governments, large multinational companies, small startups, doctors, hospitals, patients, and caretakers. Of course, depending on the type of BM and corporate goals, customer segments and communication channels with them may be different. In the medical devices industry and biotechnology, from the viewpoint of outbound open innovation BMs, groups of customers may include a wide spectrum ranging from large multinational companies to small and medium startups and to even large and leading industries. In fact, customers may be companies that seek to outsource their R&D findings [43]. Furthermore, in inbound open innovation BMs, customers may come from any stratum of the society or any market that seeks innovation, initiatives, and efficiency in a product. Such customers may be larger, international, pharmaceutical companies, regional distributors, patients, professional hospitals, doctors, and even governments [20]. Sustainable BMs, contrary to the other models, do not exclusively care about consumers and costumers, as they try to find environmentally and socially concerned market segments. In fact, sustainable BMs, instead of targeting needs, focus on customers’ logic [44].

Dynamic BMs are shaped as a result of constant communication with customers, and taking into account their needs, opinions and demands. Receiving continuous feedback from customers, firms will be able to change their direction or improve their strategy. Importantly, in dynamic BMs, even customer segments too can undergo changes in time due to various reasons such as a change of taste or market needs [1]. Spin-off BMs target a market segment that seeks modern, high-tech value propositions. In the field of health, the market segment may include large international pharmaceutical companies, distributors, patients, professional hospitals, foreign governments, or any social segment that seeks to reach unique, innovative, and technologic value propositions [13]. High-tech-low-fee BMs, too, normally focus on emerging markets where cost shapes a significant factor for customers. In such markets, customers may be hospitals, clinics, and doctors in less developed regions/villages, governments from emerging economies, and NGOs [34]. In HIT-ECM BMs, customer communications take place through marketing and generating “appropriate content” for different beneficiaries in all commercial operations of a given product/service. Such models use social media as efficient tools for communicating with customers and establishing customer segments [33]. In back-end BMs, customers are divided into several groups. First there are general and ordinary customers who may not be seen as potential sources of profit to the firm. The second group includes organizations and institutions that regard the firm's support activities as a value. These customers may be research organizations, governments, and universities [32].

#### 4.1.4 Finances

In terms of finance, outbound open innovation BMs receive the revenue arising from collaborative services on the spot, but given the project-based nature of the work, the revenue is not usually recurrent. Nonetheless, in inbound open innovation BMs, costs may be paid on the spot to the collaborating companies involved, although the revenue may be gained with some delay [43]. Unlike the other BMs, sustainable BMs do not limit revenue only to the creation and capture of economic value. Instead, they as much as possible try to reduce cost, loss/waste, the consumption of raw materials, and environmental impacts. Sustainable BMs normally emphasize products that require less materials [16]. Dynamic BMs, by adjusting the firm to market needs, enable the firm to timely predict and trace customer changes, which is a strategy guaranteeing the source of income over time. The important issue, however, is that in such BMs sources of income may undergo changes through cause-effect cycles as well as changes in products, the value proposition, customer segments, and other similar factors. Because these BMs work under the influence of other factors and the environment, they are able to make timely changes [41]. In dual BMs, as the system simultaneously concentrates on both innovation and efficiency, it can have various sources of revenue. The source of income arising from innovation involves higher levels of risk, but instead it can support the financial needs of innovation through efficiency [40]. In spin-off BMs, profitability is gained based on cost advantage in international markets and tax advantages. These BMs can potentially gain profit through research-based partnership, tax advantages, product design, and research projects [20].

In high-tech-low-fee BMs, value capture and financial issues reflect value creation activities in the company in terms of financial costs and revenue. Their value capture procedure rests on a philosophy that suggests if the product and the commercial model are sufficiently low-cost, a good profit margin can be gained. In such BMs, besides traditional and ordinary BMs, financial schemes can operate based on pay-per-use, software services, or leasing to compensate for the financial limitations of customers [11]. Gaining revenue in back-end BMs, contrary to the other models, is divided into two distinct parts: (a) the frontline of business which is responsible for delivering goods to customers; companies may not even expect to gain profit from this section; and (b) the backline which is normally responsible for the main revenue and the profitability process of the company. In these BMs, the mainstream income of the company is gained from the value proposition through support activities offered to firms, organizations, or even governments [32]. Finally in the case of PSS BMs, revenue is usually obtained through service units or product operations offered to customers (e.g. joint services, pay-per-use) [35].

### 4.2 BMs used in health-tech

In this section, the specifications of the BMs as mentioned in the papers selected are investigated as case studies in the market of medical devices (biotechnology). This section addresses legal conditions, methods of financial supply, target groups, and product types. In the 34 papers selected, a total of 52 business-related case studies were reported in various companies in healthcare, medicine, biotechnology, and high-tech industries. Given the nature of the present study, most of the companies were innovative and knowledge-based.

Furthermore, the target customers of companies engaged in medical devices or biotechnology could be broken into some groups based on the activity type and the BM used: (a) end users: this category includes patients, or part of people in the society as the ultimate target market; (b) intermediate customers: this groups included doctors, hospitals, clinics, paraclinics, healthcare centers, and similar entities; such customers had a mediatory position between medical services and target customers (e.g. patients or other people in the society); and (c) wholesale customers: this group involved larger companies, academic or non-profit institutions, regional/international distributors, governments, etc. These two last groups do not tend to directly provide services to end users, but only facilitate the process.

In terms of finance, the companies under investigation drew on diverse methods: governmental subsidiaries or financial aids, tax advantages, science and technology parks, and governmental research grants were the most remarkable factors in this regard. Considering the fact that knowledge-based and R&D activities would be very costly in the initial stages of a product's lifespan and would generate little profit, public or governmental budget for R&D can serve as an overriding tool for reducing private costs of a R&D project and for turning a non-profitable project to a profitable one. Such budges would reduce fixed costs in R&D projects and increase their change of success. Furthermore, collaboration and partnership with large companies, joint investment, joint contracts, strategic partnerships, and the use of various forms of the private sector's budgetary consortiums were among the activities associated with financial supply in the companies under investigation. As far the legal issues were concerned, the registration of property rights and patents were addressed by the companies. One of the issues raised by the majority of medical companies was obtaining the required permits from the US Food and Drug Administration and the Department of Health and Human Services, as well as market certifications in Europe and Canada. Moreover, obtaining quality-related certifications from internationally reliable companies (e.g., ISO) were some of the other concerns mentioned. Importantly, many of the papers emphasized that invention patent rights must contain the most detailed aspects of business and not merely technical issues.

### 4.3 Exploring uncertainty in BMs of health-tech

Since this article focuses on companies that are naturally based on innovation and knowledge development, special attention to the causes of uncertainty in business is critical because business innovation is intrinsically tied to uncertainty and its management requires particular understanding. Knowledge-based businesses are involved with more uncertainty and dynamism, so any BM that is chosen must be able to face the challenges of uncertainty properly. In business, uncertainty can be defined as any unpredictable event that disrupts a company's performance [61]. The business environment in the field of medical devices, biotechnology and high-tech industries, which actually are knowledge-based companies, due to the disruption caused by new digital technologies, deregulation, new BMs, the threat of new competitive companies, inventions, discoveries and the inherent dynamics of knowledge is increasingly dynamic and uncertain [25].

**Fig. 2:**
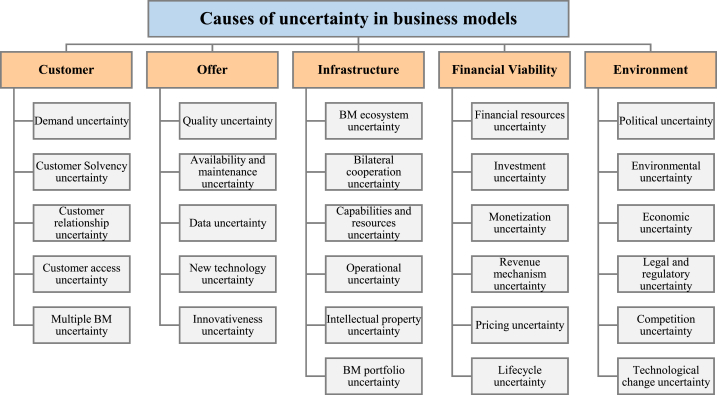
The framework of the causes of uncertainty in BMs.

With an in-depth literature review, an effort was made to provide a framework for medical devices, biotechnology, and high-tech industries to recognize and understand the roots and causes of uncertainty in different areas of their business models. This framework, which includes 28 groups of BM uncertainty factors, is shown in Fig. 2. The goal is to provide a clear insight into the uncertainties in different parts of BM so that appropriate advanced warnings are available for companies in this field to limit multiple surprises. The proposed framework has practical significance for companies to engage in business ecosystem creation in uncertain and dynamic environments to create and absorb value from emerging complex innovations. This framework can also be used to identify and assess risk and uncertainty factors in existing BMs. Adapting the design of a BM to overcome some risk and uncertainty factors can lead to new innovations and developments. As illustrated in Fig. 2, the causes of business model uncertainty were categorized into six aspects: customers, offers, infrastructures, financial capability, and the environment [62].

In the customers category, uncertainty factors refer to all critical aspects of BM's potential customers. It relates to factors related to customer relationship, customer solvency, customer access, and factors related to the performance of multiple BMs as well as customer demand. In the field of medical devices, biotechnology and high-tech industries, the dynamics of innovations, inventions, and knowledge production lead to the dynamics of demand and trends. Innovations fuel the creation of demand and its dynamics. It makes forecasting demand difficult. Considering the speed of innovations and knowledge developments in this field, companies must be flexible and react quickly. In this section, factors related to misinterpretation or failure to meet customer demand can include a lack of focus on customer needs, failure to attract or retain customers, or failure to meet society's needs adequately. In addition, companies may make mistakes in the credit rating of customers, which is in the field of factors related to customer debt payment. Factors that can damage customer relationships include loss of customer relationships, opportunistic or unfavorable customer behavior, or inflexible agreements. Factors that hinder customer access can also include lack of access to the market, lack of a strong intermediary, challenges and barriers to entering the market, or strong competitors. Also, aspects attributed to the emergence of multiple BMs in an organization can cannibalize the existing customer base, lose loyal customers, or offend existing customers, leading them to become a new competitor [25].

The second category is uncertainty factors that jeopardize the value proposition. These include uncertainty arising from the quality of the offer (i.e., offer quality, its new technology or innovation), availability and maintenance, data, (new) technology and innovation. Aspects related to the quality of an offer include the gap in expected performance versus delivered performance, durability, and performance. Uncertainty in availability and maintenance has critical aspects related to the availability and maintenance of an offer as an important component of the offer value and the consequences related to poor performance of the offer. Uncertainty related to the use of data such as data security, data ownership, data privacy and data quality is another important aspect of risk and uncertainty in the BMs of innovative companies in this field. This can be related to digital BMs that collect and use data or sell their goods online. Another important group of offer risk factors is related to the availability and maintenance of offer value. This can happen in a BM that provides rental services. Two critical value proposition components for these types of BMs are the availability and trouble-free operation of rental services, guaranteed by regular maintenance. Possible risk events and uncertainty arising from these types of factors are customer dissatisfaction, which can potentially lead to customer churn, reduced profitability, and image loss [61].

The third category of uncertainty causes covers all aspects of the BM infrastructure. In the field of medical equipment, biotechnology and high-tech industries, infrastructures also focus on methods of knowledge production and commercialization. Knowledge is the basic pillar of these companies. The key resources in these companies are prominent scientists, faculty and inventors. In addition to factors related to the business ecosystem, value network, and partnerships in a BM, as well as critical capabilities, resources, and intellectual property, this grouping includes operational risk factors and uncertainty related to human behavior or technical errors (such as equipment failure). These uncertainty factors can be critical for manufacturing companies, such as those that produce goods in a chain consisting of several interdependent stages. This is important in medical devices, biotechnology and high-tech manufacturing companies because potential risks and uncertainty events like this can cause bottlenecks, delays or defaults in the process and thus jeopardize the delivery of the value proposition [62]. The fourth category refers to the causes of uncertainty in the field of financial capability, which includes factors related to financial resources, costs, revenue generation, and the revenue model of a BM. These factors include all aspects that affect the ability of the BM manager to finance the BM. Risk factors and uncertainty about high investments are also part of this category. Risk factors and investment uncertainty can be relevant if high initial investments or capital ties are necessary when starting a new BM or when an existing BM is very capital intensive. In medical devices, biotechnology and healthcare sector, incomes are sometimes realized with delay and sometimes not repeated [25]. On the other hand, the success of a scientific or research project can turn into a long-term financial flow. Finally, the category of uncertainties related to the external environment includes aspects such as risk factors and political, environmental, and economic uncertainty, as well as risk factors and uncertainty of competition or technological changes. It can also include natural disasters that damage or destroy a production site. Such events can disrupt the BM or even the entire business ecosystem by harming suppliers or ecosystem partners [63].

### 4.4 A dynamic sustainable business model proposition for health-tech

Healthcare, medical devices, and biotechnology are crucial industries driven by innovation and technology. Yet, these sectors face dynamic challenges due to disruptive innovations, changing patient needs, strict regulations, sustainability concerns, and uncertainties. To tackle these issues, a literature review reveals the need for dynamic and sustainable business models. Providing a sustainable and dynamic business model for the Healthcare, Medical Devices, and Biotechnology Industries is of paramount importance due to several compelling reasons. In a rapidly evolving landscape driven by technology, a dynamic model is essential to seamlessly integrate new innovations into products and services, ensuring competitiveness and relevance. The patient-centric nature of healthcare demands a model that can quickly adapt to shifting patient needs and preferences. This personalized approach enhances patient satisfaction and outcomes. Moreover, the stringent regulatory environment requires agility for compliance. A dynamic model enables swift responses to changing regulations, ensuring uninterrupted operations. Additionally, uncertainties inherent in healthcare are compounded by market shifts and treatment advancements. A dynamic model equips companies to navigate uncertainties and capitalize on emerging opportunities.

The imperative for environmental sustainability mandates eco-friendly practices. A dynamic model facilitates the adoption of sustainable solutions, aligning with global environmental concerns. Furthermore, the complex stakeholder relationships in healthcare demand a model that accommodates diverse interests. A dynamic approach fosters collaborations, mutual growth, and a harmonious ecosystem. For sustained competitiveness, innovation is paramount. A dynamic model encourages continuous product and service enhancement, differentiation, and market positioning. Emphasizing sustainability and adaptability ensures long-term resilience. A dynamic model equips companies to withstand disruptions, economic fluctuations, and industry changes. With healthcare challenges on the rise, responsiveness is crucial. A dynamic model enables quick recalibration of efforts to address urgent healthcare needs effectively. In summary, the integration of technological dynamism, patient-centricity, regulatory agility, innovation, sustainability, stakeholder engagement, and crisis responsiveness underscores the necessity of a sustainable and dynamic business model. It empowers companies to excel, innovate, and positively impact the evolving Healthcare, Medical Devices, and Biotechnology Industries.

**Fig. 3:**
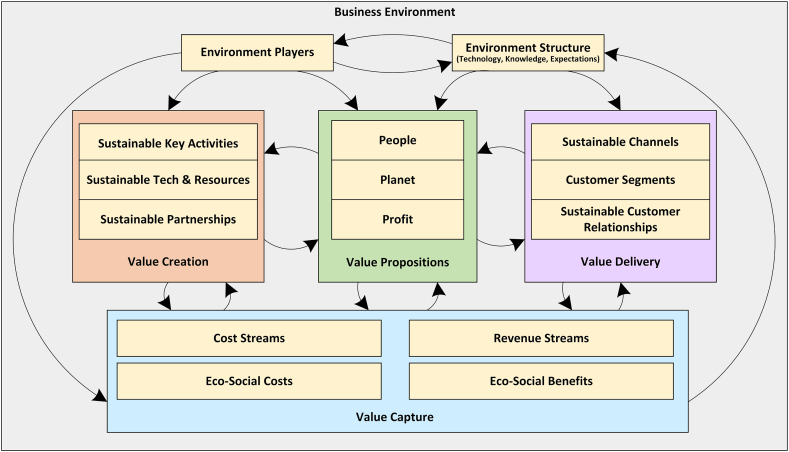
Visual representation of the DSBM for health-tech.

For these reasons, the Dynamic Sustainable Business Model (DSBM) for the Healthcare, Medical Devices, and Biotechnology Industries (DSBM for Health-Tech) is proposed, aiming to promote agility, sustainable innovation, and long-term success. Unlike conventional static approaches, the model emphasizes dynamism, adaptability, and sustainability as cornerstones for success. It acknowledges the interconnectedness of stakeholders and variables in the Health-Tech ecosystem, enabling strategic responses to evolving circumstances while considering economic, environmental, and societal impacts in decision-making. The proposed DSBM for Health-Tech is shown in Fig. 3.

The initial visual model, as illustrated in Fig. 3, presents a comprehensive depiction of the Designing Sustainable Business Model (DSBM) for Health-Tech. This model is designed to address the intricate dynamics of the healthcare, medical devices, and biotechnology sectors while prioritizing sustainability and adaptability. At its core, the model comprises five interconnected dimensions: Business Environment, Value Propositions, Value Creation, Value Delivery, and Value Capture. These dimensions collectively create a holistic framework that enables health-tech businesses to thrive in a constantly evolving landscape.

The Business Environment dimension encompasses a dynamic ecosystem that involves a multitude of players and stakeholders. This includes academic institutions, research organizations, startups, industry partners, healthcare providers, patients, government bodies, investors, regulatory agencies, distributors, suppliers of sustainable materials, insurers, and societal stakeholders. The environment is shaped by rapidly evolving technology, knowledge dissemination, and dynamic expectations, forming a foundation that drives continuous innovation and adaptation. Value Propositions, the second dimension, revolves around the harmonious integration of People, Planet, and Profit. People Values emphasize patient-centricity, personalized healthcare solutions, and overall well-being. Planet Values underscore eco-friendly practices, circular economy principles, and sustainable product design to minimize environmental impact. Profit Values prioritize financial sustainability and growth, aligned with ethical business practices and responsible resource allocation. The Value Creation dimension is rooted in the integration of Sustainable Key Activities, Sustainable Tech & Resources, and Sustainable Partnerships. These elements combine to deliver innovative and environmentally conscious healthcare solutions. Sustainable Key Activities involve continuous innovation, adaptable R&D, and circular economy practices, ensuring ongoing value creation while minimizing environmental impact. Sustainable Tech & Resources leverage advanced technologies and eco-friendly resources to develop innovative medical solutions, promoting sustainability. Sustainable Partnerships involve collaboration with various stakeholders to foster innovation and drive sustainable healthcare advancements.

The fourth dimension, Value Delivery, is focused on efficiently distributing innovative healthcare solutions. This occurs through direct sales, online platforms, partnerships with distributors, and active participation in industry events. These channels ensure timely access for patients, healthcare providers, and stakeholders. The model emphasizes sustainable channels, diverse customer segments, and lasting relationships that prioritize patient-centric care and environmental responsibility. Finally, the Value Capture dimension involves generating revenue through various streams, such as product sales, licensing, services, and subscriptions. This revenue is aligned with positive societal and environmental impacts. The dimension balances cost streams, revenue streams, eco-social costs incurred by business operations, and the eco-social benefits created by the model. This approach ensures a sustainable and ethical financial foundation. Overall, the DSBM for Health-Tech is depicted as a comprehensive and interconnected model, encompassing various dimensions and components that collectively enable health-tech businesses to thrive in a dynamic and sustainable manner. This model is rooted in adaptability, innovation, and a deep commitment to addressing both healthcare needs and environmental considerations. As illustrated in the visual representation provided in Fig. 3, the Designing Sustainable Business Model (DSBM) for Health-Tech comprises a comprehensive framework that encompasses various components, each contributing to the model's holistic approach to healthcare innovation and sustainability. These components and their detailed descriptions can be found in Table 4, offering insights into the key aspects of the DSBM for Health-Tech.

**Table 4:** Components and descriptions of the DSBM for health-tech.

Main Components		Sub Components	
Business Environment	The environment of the DSBM for Health-Tech encompasses a dynamic ecosystem comprising academic collaborations, industry partnerships, regulatory influences, societal stakeholders, and innovative technology suppliers, all contributing to a holistic approach to healthcare innovation and sustainability.	Environment Players	The main players and stakeholders in the environment include academic institutions, research organizations, startups, industry partners, healthcare providers, patients, government bodies, investors, regulatory agencies, distributors, suppliers of sustainable materials, insurers, and societal stakeholders.
		Environment Structure	The Environment Structure incorporates rapidly evolving technology, cutting-edge knowledge dissemination, and dynamic expectations, creating a foundation that drives continuous innovation and adaptation.
Value Propositions	The Value Propositions in the DSBM for Health-Tech encompass the harmonious integration of People, Planet, and Profit, offering high-tech medical solutions that prioritize superior patient outcomes, environmental sustainability, and economic growth.	**People Values**	The People Values within the DSBM for Health-Tech emphasize patient-centricity, personalized healthcare solutions, and the well-being of individuals and communities.
		**Planet Values**	The Planet Values underscore the integration of eco-friendly practices, circular economy principles, and sustainable product design to minimize environmental impact.
		**Profit Values**	The Profit Values focus on achieving financial sustainability and growth while aligning with ethical business practices and responsible resource allocation.
Value Creation	The value creation in the DSBM for Health-Tech stems from the integration of Sustainable Key Activities, leveraging Sustainable Tech & Resources, and fostering Sustainable Partnerships to deliver innovative and environmentally conscious healthcare solutions that prioritize patient-centricity, environmental responsibility, and long-term economic viability.	**Sustainable Key Activities**	Sustainable Key Activities encompass continuous innovation, adaptable R&D, and circular economy practices, ensuring ongoing value creation while minimizing environmental impact.
		**Sustainable Tech & Resources**	The Sustainable Tech & Resources aspect in the DSBM for Health-Tech entails leveraging advanced technologies and eco-friendly resources to develop innovative medical solutions while promoting environmental sustainability.
		**Sustainable Partnerships**	The Sustainable Partnerships component involves collaborating with academic institutions, research organizations, startups, and industry players to pool diverse expertise, fostering innovation and driving sustainable healthcare advancements.
Value Delivery	The Value Delivery aspect involves efficient distribution of innovative healthcare solutions through direct sales, online platforms, strategic partnerships, and participation in industry events, ensuring timely access for patients, healthcare providers, and stakeholders. It ensures sustainable channels, reaching diverse customer segments, and nurturing long-lasting, ethical, and personalized relationships that prioritize patient-centric care and environmental responsibility.	**Sustainable Channels**	Sustainable Channels encompass direct sales, online platforms, partnerships with distributors, and active participation in industry events, all tailored to effectively engage stakeholders while maintaining environmental consciousness.
		**Customer Segments**	The Customer Segments encompass patients seeking advanced medical solutions, healthcare providers interested in cutting-edge technologies, government agencies seeking cost-effective options, and impact-driven investors looking to support innovative healthcare projects.
		**Sustainable Customer Relationships**	The Sustainable Customer Relationships involve personalized support for healthcare providers, continuous feedback loops with patients for ongoing improvements, transparent and ethical communication with stakeholders, and the cultivation of long-term partnerships that foster mutual growth and innovation.
Value Capture	Value capture in the DSBM for Health-Tech is realized by generating revenue through diverse streams, including product sales, licensing, services, and subscriptions, while aligning financial success with positive societal and environmental impacts. The Value Capture in the DSBM for Health-Tech encompasses the balance between cost streams, revenue streams, eco-social costs incurred by the business's operations, and the tangible and intangible eco-social benefits created by the business model, ensuring a sustainable and ethical financial foundation.	**Cost Streams**	The Cost Streams in the DSBM for Health-Tech involve the allocation and management of financial resources required for sustainable operations, innovation, and compliance within the healthcare, medical devices, and biotechnology sectors including production, R&D, marketing, and staffing.
		**Revenue Streams**	The Revenue Streams in the DSBM for Health-Tech encompass the diversified sources of income derived from the sale of innovative medical devices, sustainable biotechnological solutions, licensing of intellectual property, and subscription-based models for data-driven healthcare services.
		**Eco-Social Costs**	The Eco-Social Costs in the DSBM for Health-Tech account for the environmental and social impacts associated with business operations, ensuring a comprehensive assessment of both positive and negative externalities focusing on resource use, waste generation, and societal implications.
		**Eco-Social Benefits**	The Eco-Social Benefits encompass the positive contributions and outcomes that the business generates for the environment and society through its sustainable practices and innovations.

The DSBM for Health-Tech thrives within a dynamic framework, employing continuous feedback loops and adaptability. This safeguards responsiveness to evolving trends, customer preferences, technological advancements, and market shifts. Integrated feedback loops span interactions with customers, stakeholders, regulatory bodies, and industry peers. Valuable insights collected therein are transformed into actionable improvements, driving enhancement in medical devices and biotech solutions, tailored to patient and healthcare provider needs.

Feedback loops' pivotal role in innovation is evident in the DSBM for Health-Tech. Through ongoing evaluations of customer experiences, market demands, and technological strides, the model ensures offerings remain cutting-edge, facilitating rapid responses to emerging opportunities. This dynamic process deeply affects Health-Tech companies' business models, fostering innovation through a culture of continuous improvement. Real-time data guides strategic decision-making for product development, marketing, and resource allocation, ensuring alignment with market dynamics. This communication with customers not only enhances relationships but also elevates brand perception, loyalty, and satisfaction. The adaptability embedded in dynamic feedback loops empowers swift responses to disruptions, uncertainties, and industry shifts. This translates into improved resilience, superior risk management, and a competitive edge, positioning Health-Tech companies as industry leaders. Central to the DSBM for Health-Tech is its embodiment of sustainability principles. Environmental, economic, and societal considerations are interwoven. Eco-friendly practices and circular economy principles minimize environmental footprints, while renewable materials and circular supply chains drive positive environmental impact.

Economic sustainability is achieved through diversified revenue streams and efficient operations. Multiple revenue models ensure financial stability even during market fluctuations. The model also upholds social responsibility, benefiting patients, healthcare providers, employees, and communities. Partnerships with academia and startups foster knowledge exchange, community engagement, and societal development. Sustainability is assured by weaving it throughout the business model. Continuous feedback loops track metrics, driving ongoing improvement and alignment with environmental, economic, and societal goals. The integration of circular practices, sustainable partnerships, and eco-friendly innovation underscores commitment to environmental preservation, economic viability, and societal progress. In essence, the DSBM for Health-Tech unites environment, economy, and society in a harmonious synergy, guaranteeing sustainability. Dynamic integration empowers responsible, forward-looking business practices, propelling healthcare innovation and sustainable advancement.

In the DSBM for Health-Tech, it is crucial to distinguish between “eco-social costs” and “cost streams,” as both play distinct roles in the business model. Eco-social Costs encompass the environmental and social impacts associated with a company's operations. They include factors like environmental degradation, resource depletion, and negative societal impacts. In the DSBM, managing eco-social costs is integral to ensuring long-term sustainability and corporate responsibility. The model addresses these costs through sustainable practices like using eco-friendly production methods and engaging in community-focused initiatives, aligning business operations with broader societal and environmental goals.

Meanwhile, cost streams represent the direct financial expenditures involved in running a business. This includes operational expenses like manufacturing costs, research and development, marketing, and employee salaries. The DSBM aims to manage these cost streams efficiently, aligning them with innovation and sustainability. Strategies like lean operations, cost-effective supply chain management, and investment in efficient technologies are employed to balance financial performance with the overarching sustainable goals of the business. In the context of the DSBM, these two cost types are interconnected yet distinct. Efficient management of cost streams directly influences a company's profitability and competitiveness, while responsibly addressing eco-social costs ensures long-term viability and aligns the business with global sustainability objectives. The DSBM framework is designed to navigate this balance, allowing companies in the healthcare, medical devices, and biotechnology industries to thrive economically while upholding their environmental and social responsibilities.

The Designing Sustainable Business Model (DSBM) for Health-Tech offers distinct differences and advantages compared to the traditional Business Model Canvas (BMC). Rooted in the Healthcare, Medical Devices, and Biotechnology Industries, the DSBM integrates sustainability, innovation, and adaptability as fundamental principles for success. Unlike the basic BMC, the DSBM places a strong emphasis on sustainability across all dimensions. It intertwines eco-friendly practices, circular economy principles, and sustainable product design, ensuring a positive environmental impact. Additionally, the DSBM introduces dynamic elements like continuous feedback loops, adaptability to uncertainties, and scenario planning. These features allow companies to remain agile and responsive in the face of rapidly changing technologies, market shifts, and regulatory landscapes. The DSBM's core revolves around innovation, collaborating with research organizations, startups, and industry partners. This encourages rapid product development and creates a culture of ongoing improvement. The model integrates the triple bottom line approach—People, Planet, Profit—into its value propositions, promoting balanced consideration of social, environmental, and economic factors.

Advantages of the DSBM are substantial. Companies adopting this model position themselves as sustainability leaders, attracting stakeholders aligned with ethical practices. The dynamic nature of the DSBM fosters accelerated innovation, helping companies develop cutting-edge solutions tailored to evolving customer needs. Agility and adaptability become cornerstones of competitiveness, enabling businesses to navigate market shifts with resilience. Long-term viability is another strength of the DSBM, as its emphasis on sustainability and innovation ensures companies remain relevant through technological cycles and uncertainties. Societal impact is positive, with collaborations and partnerships extending to healthcare providers, academic institutions, and local communities. The DSBM's eco-friendly practices align with global sustainability goals, reducing the environmental footprint of business operations. Furthermore, the DSBM aids companies in complying with changing regulations through continuous monitoring and dynamic adaptation. This prevents regulatory setbacks and positions companies as compliant industry leaders. In essence, the DSBM for Health-Tech offers a holistic and forward-looking approach to business. By integrating sustainability, innovation, and adaptability, this model equips companies to thrive in a rapidly changing landscape. The result is a positive impact on healthcare outcomes, environmental sustainability, and long-term business success.

Finally, DSBM for Health-Tech is designed to effectively manage uncertainties in the Healthcare, Medical Devices, and Biotechnology Industries. It achieves this through proactive scenario planning, adaptability, continuous feedback loops, collaborations, strategic resource allocation, sustainability integration, inclusive value propositions, and risk management. These features transform uncertainties into opportunities for preparedness, innovation, responsiveness, and growth. The DSBM empowers companies to navigate changing circumstances with agility, leveraging data-driven insights and external partnerships. By embracing sustainability and inclusive strategies, the model equips businesses to effectively address uncertainties and turn them into positive outcomes, contributing to long-term success and impactful innovation in healthcare.

## 5 Conclusions

This systematic literature review illuminates the intricate landscape of business models for innovation and value creation within the medical, biotechnology, and healthcare spheres. Through meticulous analysis of scholarly articles and industry cases, this research unveils a taxonomy of nine prevalent business models, including open innovation BMs, Sustainable BMs, Dynamic BMs, Dual BMs, Spin-offs BMs, Frugal BMs, high-tech entrepreneurial content marketing BMs, Back-end BMs, Product-service systems BMs. The comparative analysis of these models across four critical dimensions - infrastructure, offering, customers, and finances - has cast light on their distinct intricacies and strategic orientations. Among these models, open innovation, sustainability, and dynamicity emerged as foundational pillars, providing an essential underpinning for the formulation of effective business approaches. The study illuminated the dynamic interplay of these models, where their integration leads to nuanced and potent strategies for navigating the intricate landscape of medical devices, biotechnology, and healthcare industries.

Moreover, the research addressed the inevitable specter of uncertainties that pervade the business landscape of these sectors. By conceptualizing a comprehensive framework encompassing 28 groups of uncertainty factors, the study offered a systematic approach to anticipate, decipher, and mitigate risks arising from technological shifts, regulatory dynamics, financial fluctuations, and environmental contexts. This framework stands as a beacon guiding health-tech enterprises towards informed decision-making in the face of multifaceted uncertainties. Culminating at the zenith of this research was the proposition of the DSBM for Health-Tech. This innovative model, intertwining adaptability, continuous enhancement, and sustainability, epitomizes a holistic approach tailored to the unique demands of the medical, biotechnology, and healthcare domains. By fostering an iterative feedback loop, a responsive posture towards change, and an unwavering commitment to ethical and societal well-being, the DSBM for Health-Tech paves the path for business models that not only navigate the intricacies of innovation but also seamlessly intertwine them with sustainability imperatives.

This research has yielded significant contributions spanning healthcare, medical devices, biotechnology, and business management. By meticulously analyzing and comparing various business models, it offers profound insights into strategies tailored for negotiating the intricacies within these sectors. One prominent accomplishment lies in the formulation of a comprehensive framework outlining 28 groups of uncertainty factors in business models. This framework equips companies with practical tools to effectively manage a spectrum of challenges, ranging from technological shifts to regulatory dynamics, financial uncertainties, and broader environmental considerations. Central to this research is the development of the DSBM for Health-Tech. This adaptable model seamlessly integrates innovation, sustainability, and stakeholder engagement, effectively aligning business operations with ethical values and ecological imperatives. Moreover, the practical implementation of sustainability principles within the DSBM caters to the pressing need for environmentally responsible solutions. Additionally, this study addresses a significant gap in the current literature. It is the first to present a comprehensive literature review and meta-analysis focusing specifically on business models within the healthcare, medical devices, and biotechnology industries, particularly in the context of managing inherent uncertainties. This unique contribution provides substantial insights, enriching both academic research and practical applications in these fields.

Concurrently, the research's insights into risk mitigation strategies empower companies to adeptly navigate the intricate landscape of uncertainties. Looking forward, this study not only serves as a stepping stone but also paves a clear path for future investigations. It invites exploration into various facets, such as case studies, dynamic sustainability practices, refined risk management techniques, enhanced stakeholder engagement, and the ethical dimensions associated with these industries. In conclusion, this research seamlessly integrates innovation, business models, sustainability imperatives, and the management of uncertainties. The culmination of its insights enables businesses to take informed strides towards fostering positive impacts and achieving sustainable growth within these vital sectors.

Looking ahead, the research opens up compelling avenues for future exploration. Directing attention towards the practical implementation of the DSBM for Health-Tech and assessing its performance across varied contexts holds immense promise. Concurrently, deeper investigations into integrating dynamic sustainability within business models could uncover strategies for harmonizing adaptability and responsible practices. Tailored risk management approaches specific to the healthcare, medical devices, and biotechnology sectors should be explored to mitigate uncertainties effectively. Furthermore, delving into stakeholder engagement dynamics, understanding the influence of emerging technologies, and addressing ethical dimensions of innovation stand as crucial focal points. In summary, the research presents a roadmap for empirical validation, nuanced sustainability integration, risk management, stakeholder engagement dynamics, technology's role, and ethical considerations in shaping future research endeavors.

In discussing the applicability of the study's findings to firms in various countries, the global significance of the healthcare, medical devices, and biotechnology industries was acknowledged. Despite variations arising from different economic development stages and government policies, these industries face common challenges and trends that transcend national borders, such as rapid technological advancements and universal health concerns. The research provides a foundational understanding of business models that, while informed by a broad analysis, can be adapted to local contexts. It is recognized that economic development and policy environments shape business practices; however, the core principles of innovation and strategic management in these industries have a universal relevance. By presenting adaptable and flexible business models, the findings offer a framework that firms can tailor to align with their specific national conditions, including economic status and regulatory frameworks. This approach allows for the nuanced application of the insights, making them valuable for firms operating in diverse global markets, each with their unique challenges and opportunities.

## Funding

The work was supported by the internal project “SPEV – Economic Impacts under the Industry 4.0/Society 5.0 Concept” and Excellence 2022, University of Hradec Králové, Faculty of Informatics and Management, Czech Republic. At the same time, the authors would like to acknowledge the support provided by the Economic and Management College of 10.13039/501100004193Nanjing University of Aeronautics and Astronautics, China.

## Additional information

No additional information is available for this paper.

## CRediT authorship contribution statement

**Ehsan Javanmardi:** Writing – review & editing, Writing – original draft, Resources, Methodology, Investigation, Data curation. **Petra Maresova:** Writing – original draft, Validation, Supervision, Project administration, Investigation, Funding acquisition, Data curation, Conceptualization. **Naiming Xie:** Writing – review & editing, Writing – original draft, Validation, Software, Data curation. **Rafał Mierzwiak:** Visualization, Methodology, Formal analysis, Data curation.

## Declaration of competing interest

The authors declare that they have no known competing financial interests or personal relationships that could have appeared to influence the work reported in this paper.
